# Exploring the Cation Regulation Mechanism for Interfacial Water Involved in the Hydrogen Evolution Reaction by In Situ Raman Spectroscopy

**DOI:** 10.1007/s40820-023-01285-1

**Published:** 2023-12-18

**Authors:** Xueqiu You, Dongao Zhang, Xia-Guang Zhang, Xiangyu Li, Jing-Hua Tian, Yao-Hui Wang, Jian-Feng Li

**Affiliations:** 1 School of Ocean Information Engineering, Fujian Provincial Key Laboratory of Oceanic Information Perception and Intelligent Processing, Jimei University, Xiamen, 361021 People’s Republic of China; 2 State Key Laboratory of Physical Chemistry of Solid Surfaces, MOE Key Laboratory of Spectrochemical Analysis and Instrumentation, iChEM, College of Chemistry and Chemical Engineering, College of Materials, College of Energy, Xiamen University, Xiamen, 361005 People’s Republic of China; 3 Key Laboratory of Green Chemical Media and Reactions, Ministry of Education, Collaborative Innovation Center of Henan Province for Green Manufacturing of Fine Chemicals, College of Chemistry and Chemical Engineering, Henan Normal University, Xinxiang, 453007 People’s Republic of China; 4 Innovation Laboratory for Sciences and Technologies of Energy Materials of Fujian Province (IKKEM), Xiamen, 361005 People’s Republic of China

**Keywords:** In situ Raman, Interfacial water, Hydrogen evolution reaction, Cations

## Abstract

Interfacial water molecules are the most important participants in the hydrogen evolution reaction (HER). Hence, understanding the behavior and role that interfacial water plays will ultimately reveal the HER mechanism. Unfortunately, investigating interfacial water is extremely challenging owing to the interference caused by bulk water molecules and complexity of the interfacial environment. Here, the behaviors of interfacial water in different cationic electrolytes on Pd surfaces were investigated by the electrochemistry, in situ core–shell nanostructure enhanced Raman spectroscopy and theoretical simulation techniques. Direct spectral evidence reveals a red shift in the frequency and a decrease in the intensity of interfacial water as the potential is shifted in the positively direction. When comparing the different cation electrolyte systems at a given potential, the frequency of the interfacial water peak increases in the specified order: Li^+^  < Na^+^  < K^+^  < Ca^2+^  < Sr^2+^. The structure of interfacial water was optimized by adjusting the radius, valence, and concentration of cation to form the two-H down structure. This unique interfacial water structure will improve the charge transfer efficiency between the water and electrode further enhancing the HER performance. Therefore, local cation tuning strategies can be used to improve the HER performance by optimizing the interfacial water structure.

**Supplementary Information:**

The online version contains supplementary material available at 10.1007/s40820-023-01285-1.

## Highlights

- In situ Raman spectroscopy study of the cation regulation for interfacial water on Au@Pd core–shell nanoparticle surface during hydrogen evolution reaction process.
- Direct spectral evidence reveals new strategies to optimize the structure of interfacial water by the adjusting of radius and valence of the cation in the electrolyte.
- The optimized structure of interfacial water will improve the charge transfer efficiency between the water and electrode increasing the performance of the hydrogen evolution reaction.

**Supplementary Information:**

The online version contains supplementary material available at 10.1007/s40820-023-01285-1.

## Graphical abstract

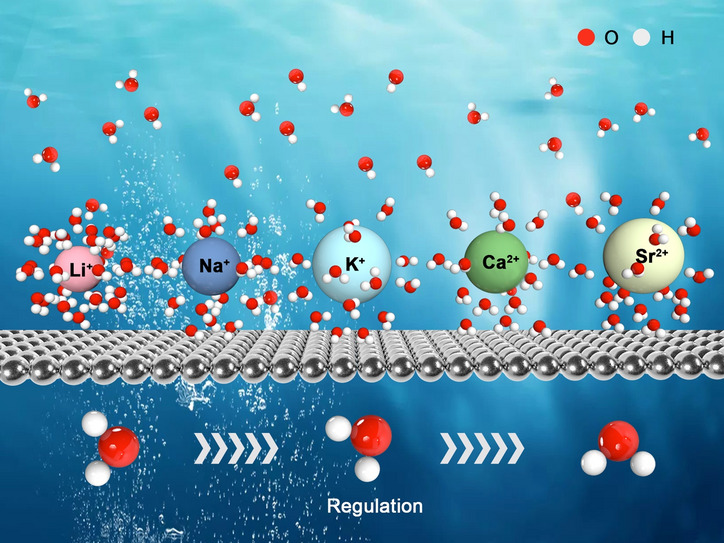

## Introduction

Water is essential for life and plays a crucial role in chemistry, biology, and materials science [1–5]. In aqueous electrocatalytic systems, water molecules and ions together constitute a significant portion of the electrode/electrolyte interface [6–9]. Many studies show that metal cations in the electrolyte have a strong impact on the electrocatalysis. Koper et al. [10–12] studied the effect of electrolyte ions on the reaction rates of a series of electrocatalytic reactions, such as hydrogen evolution reaction (HER), oxygen reduction reaction, and CO_2_ reduction reaction. Shao-Horn et al. [13] found that a series of structure-making/breaking cations in the electrolyte altered the kinetics of hydrogen evolution and oxidation reactions by up to 2 orders of magnitude. According to these findings, the interaction between interfacial water and ions causes structural changes in the interfacial water that directly impact interfacial reaction processes, thereby influencing electrocatalytic performance. Meanwhile, hydrogen bond (HB) networks, involving both ions and water molecules, form at the electrode/electrolyte interface owing to the strong dipole moment of water [14–17]. The presence of strong electric fields can induce local perturbations [18, 19] that influence the interactions between water molecules and cations at the interface. Gaining a comprehensive understanding of the structure and dynamics of interfacial water and their interactions with various cations at the molecular level has been proved challenging, and remains an ongoing pursuit in the fields of electrocatalysis and surface science.

Previous in situ spectroscopy could provide some information about the microscopic structure and dynamics of water and cations at electrode/electrolyte interface [20–28]. For example, Shao et al. [20] studied the pH-dependent changes of interfacial water on Pt surfaces using surface-enhanced infrared absorption spectroscopy. Surface sum frequency generation (SFG) is also a sensitive technique to study the interfacial water [21]. Benderskii et al. [22] found an asymmetric response of interfacial water on graphene surface under electric fields. Our group [1, 29] carried out shell-isolated nanoparticle-enhanced Raman spectroscopy to probe the behavior of interfacial water on single-crystal surfaces, revealing the key factor of hydrated cations in HER. However, the instrumentation can be prohibitively expensive, and these studies have typically focused on probing water–cation interactions. Despite past research, details about the synergistic effect between the cations and interfacial water molecules, especially the structural changes under different cation species and their corresponding valence states under HER potentials are lacking, which limit our current understanding of the mechanism of HER.

**Fig. 1:**
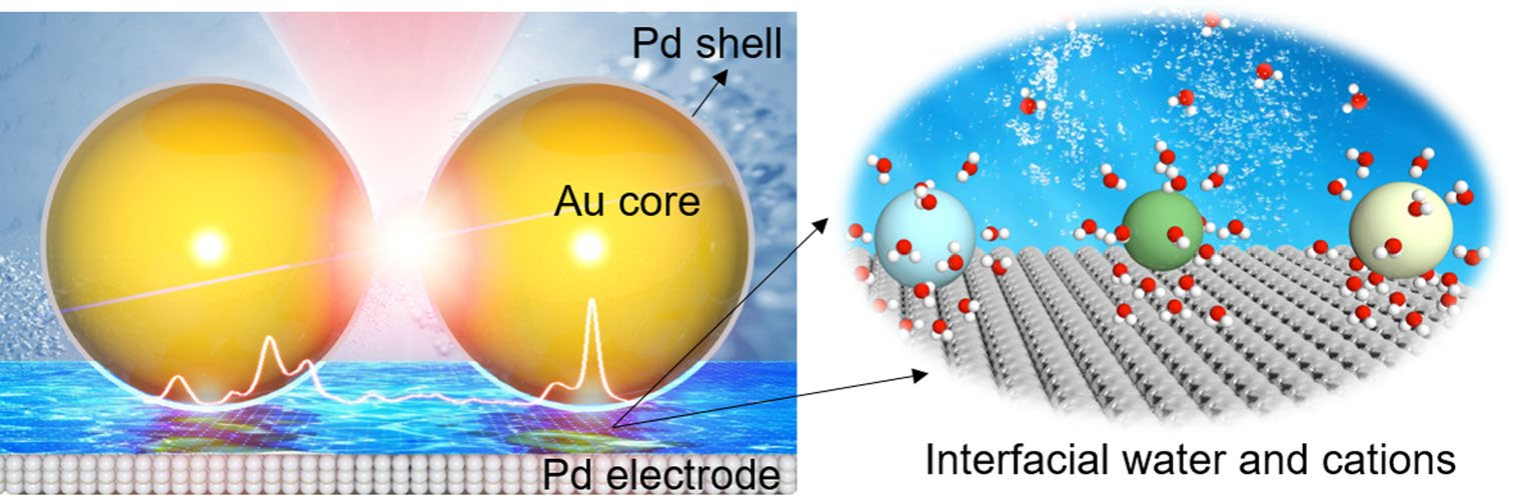
Schematic of the Au@Pd core–shell nanostructure for in situ study of interfacial water

In this paper, we constructed Au@Pd core–shell nanoparticles (NPs) and obtained electrochemical surface-enhanced Raman scatting (SERS) spectra of interfacial water in electrolytes with different cations on Pd electrode surface. Using a strategy of “borrowing” SERS enhancements from Au NP surface deposits (Fig. 1), we elucidate the effect of the potential, cation type, and electrolyte concentration on the SERS spectra of interfacial water. The population of cations relative to the interfacial water and the overall performance of the HER showed the same trend where Li^+^ < Na^+^ < K^+^ < Ca^2+^ < Sr^2+^. By combining density functional theory (DFT) calculations, we found the synergistic effect between the cations and interfacial water molecule for the HER performance by tuning the composition of interfacial water through a cation valence state and concentration regulation strategy.

## Experiment and Calculation

### Preparation of Au NPs and Au@Pd Core–Shell NPs

First, highly mono-dispersed Au NPs were prepared following the Frens’ method [30]. In short, a chloroauric acid solution (HAuCl_4_, 200 mL, 0.01 wt%) was reduced by sodium citrate (C_6_H_5_Na_3_O_7_, 1.4 mL, 1 wt%) at 100 °C. The Au spherical particle sizes were ~ 55 nm in diameter. Then, Au NPs served as seeds in solution for the second chemical reduction of dissolved Pd metal ions allowing them to deposit onto core Au surface, forming the Pd outer-shell layer. The preparation protocol of Au@Pd NPs solution is as follows: 15 mL of a freshly prepared 55 nm Au seed solution and 8.77 mL deionized water were added to 820 μL of a 1.0 mM H_2_PdCl_6_ solution at 4 °C. Under these conditions, 410 μL of 10 mM ascorbic acid was slowly injected into the above H_2_PdCl_6_ mixture solutions. The mixture solutions are stirred for 20 min to completely reduce the H_2_PdCl_6_ to Pd. During stirring processes, the mixture solutions turned from reddish brown to dark brown indicating that the Au@Pd NPs had formed.

### Electrochemical Experiments

Electrochemical experiments were carried out on a workstation (CHI 760E, CH Instrument). A homemade three-chamber glass cell, containing a Pt wire and a Hg/HgO (0.1 M KOH) as counter and reference electrodes, was used in the electrochemistry experiments. The potential was calibrated to same scale vs. reversible hydrogen electrode (RHE, E_RHE_ = E_Hg/HgO_ + 0.059pH + 0.165). A glass bridge and glass cores were used to separate each chamber. Electrochemical pinhole tests were carried out by placing the Au NPs and Au@Pd NPs on glassy carbon electrode surface. The HER activities in different cations were investigated on Pd polycrystalline electrode surface to avoid the interference from particle aggregation on performance. The electrolyte solution employed was 0.1 M MClO_4_ + 0.01 M KOH (pH 12, M = Li^+^, Na^+^, K^+^, Ca^2+^, and Sr^2+^).

### In Situ Raman Experiments

A XploRA plus confocal microprobe Raman system (HORIBA, France, and 637.8 nm excitation light) and a homemade spectroelectrochemical cell, containing a Pt wire and a mercuric oxide electrode (0.1 M KOH, Hg/HgO) as counter and reference electrodes were used in all in situ electrochemical Raman experiments. The reference electrode was set in a single chamber with a glass bridge connecting to avoid ion interference. The electrolyte solution employed was 0.1 M MClO_4_ + 0.01 M KOH (pH 12, M = Li^+^, Na^+^, K^+^, Ca^2+^, and Sr^2+^).

### DFT and Ab Initio Molecular Dynamics (AIMD) Simulations

All simulations in this work were carried out in the Vienna Ab initio Simulation Package [31, 32]. The exchange and correlation energies were distributed by using the Perdew–Burke–Ernzerhof functional within the generalized gradient approximation. Energy cutoff of 450 eV and Methfessel Paxton method with a broadening factor of 0.1 eV are used. The convergent conditions of energy and force are set at 0.00001 and 0.02 eV. The van der Waals interaction is considered through the semi-empirical D3 dispersion correction. The three layers of a (2 × 2) supercell with a 2 × 2 × 1 Г-centered k-point sampling is used to simulate the geometrical structure. We simulate the negative potential by adding the electron in the system, while charge neutrality is achieved by compensating for uniform charge background. For AIMD simulation, A Nose–Hoover thermostat is applied to keep the temperature of the canonical ensemble at 298 K, we constructed the simulation cells with three layers of (4 × 4) and (2 × 2) slab with 40 and 10 explicit water molecules on one side of the slab and a vacuum of 15 Å.

## Results and Discussion

### Characterization of NPs

**Fig. 2:**
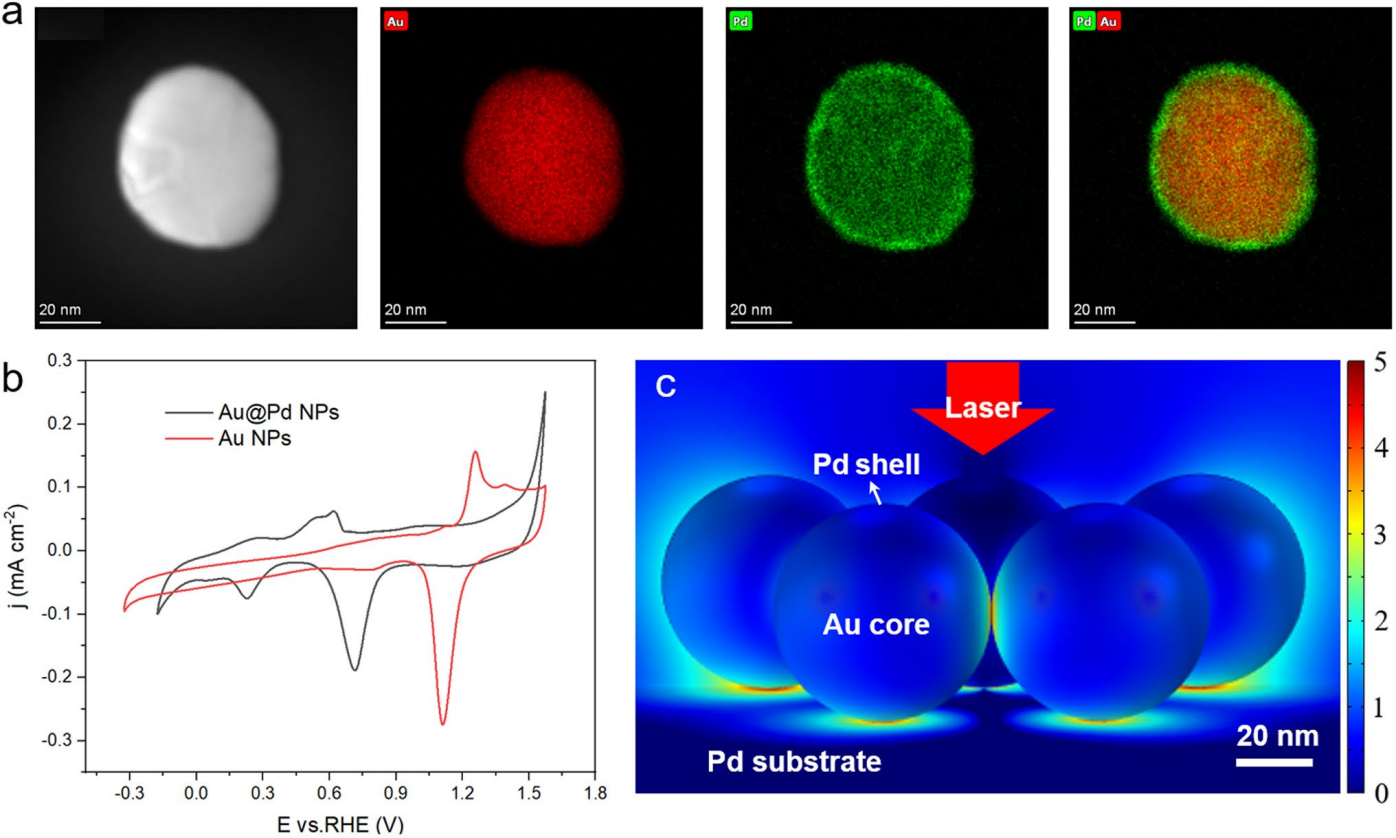
**a** TEM image and EDS elemental maps of the Au@Pd NPs. **b** CVs of Au NPs and Au@Pd NPs in 0.1 M NaClO_4_ (pH 12) with a 10 mV s^−1^ scan rate. **c** Electromagnetic field distribution of Au@Pd NPs on Pd surface

The scanning electron microscope (SEM) image (Fig. S1) of Au NPs clearly shows the typical spherical shape with a diameter of about 55 nm. Figure S2 shows the transmission electron microscope (TEM) image of Au@Pd NPs and the corresponding elemental mapping images of an Au@Pd NP are shown in Fig. 2a. Morphology and structure characterizations clearly show that the Au core is evenly surrounded by the Pd shell.

Cyclic voltammograms (CVs) were used to further characterize the Au NPs and Au@Pd core–shell NPs. Figure 2b shows a sequence of CVs for Au and Au@Pd NPs in 0.1 M NaClO_4_ saturated argon solution (pH 12). The CV of Au NPs clearly shows the Au electrooxidation/electroreduction peaks at around 1.26 and 1.11 V versus RHE [33, 34]. However, we do not observe the electrooxidation/electroreduction peaks from the CV of Au@Pd NPs, indicating that the Au NPs are densely coated with an ultrathin Pd layer. The large reduction peak at 0.71 V during the negative sweep in the CV of Au@Pd NPs is attributed the reduction of PdO according to the previous reports [35]. As the potential is shifted negatively, a modest peak at 0.23 V and a weak peak at around 0.05 V are observed due to hydrogen adsorption and hydrogen absorption, respectively [36, 37]. The hydrogen evolution reaction then proceeds at more negative potentials following adsorption and adsorption steps. During the positive sweep, a broad peak ranging from ~ 0.08 to ~ 0.39 V is visible resulting from the desorption of hydrogen. Following the hydrogen desorption process, two overlapping peaks at 0.54 and 0.62 V are observed and attributed to hydroxide adsorption on different Pd sites [38]. The electrooxidation of Pd began at 0.85 V [39], followed further oxidation and the oxygen evolution reaction.

We also used the finite element method (FEM, detailed simulation method in supplementary information) to probe the electromagnetic field distribution of Au@Pd NPs on Pd surface, modeling with a heptamer on the Pd surface. The electromagnetic field distribution results (Fig. 2c) show that the enhanced hot spot is primarily located at the contact regions between Au@Pd NPs and at the interface between Au@Pd NPs and the Pd substrate, which ensures that the Raman signals of interfacial water come from the Pd surface.

### In Situ Raman and Theoretical Simulation Results

**Fig. 3:**
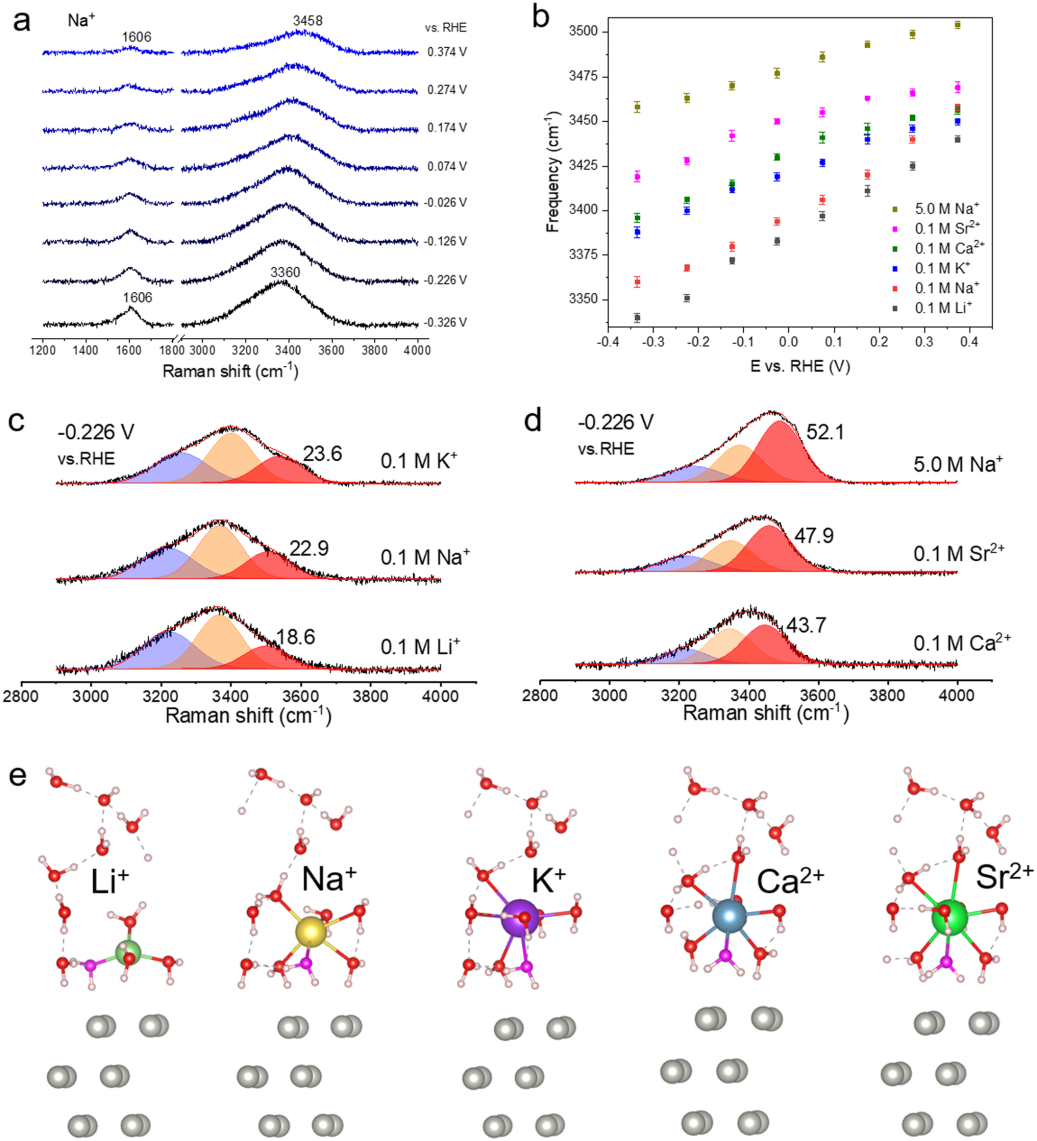
**a** In situ Raman spectra of interfacial water on Au@Pd surface in 0.1 M NaClO_4_ (pH 12) solution. **b** Plot showing the changes in Raman frequency of the OH stretching bands of interfacial water for the different cationic electrolytes. **c, d** population of interfacial water in 0.1 M MClO_4_ (pH 12, M = Li^+^, Na^+^, K^+^, Ca^2+^, and Sr^2+^) and 5.0 M NaClO_4_ (pH 12) electrolytes from in situ Raman spectra. **e** DFT calculated models of interfacial water in Li^+^, Na^+^, K^+^, Ca^2+^, and Sr^2+^ solutions on Pd (111) surface

The in situ Raman spectra of interfacial water on Au@Pd surface in 0.1 M NaClO_4_ (pH 12) solution are shown in Fig. 3a. At 0.374 V, a weak band at about 1606 cm^−1^ and a broad Raman band at about 3458 cm^−1^ attributing to the HOH bending and OH stretching modes of water, respectively, were observed [40, 41]. As the potential shifts more negative, the intensity of HOH bending and OH stretching bands gradually increase due to the aggregation of water molecules at interface. We also found that the frequency of OH stretching band is decreased from 3458 to 3360 cm^−1^ when the potential changes from 0.374 to –0.326 V. The enriched charge on the electrode surface filled the antibonding orbital of OH bond, weakened the OH bond strength, which leads to a decrease in frequency.

We further obtained the spectra of interfacial water in other cationic (Li^+^, K^+^, Ca^2+^, and Sr^2+^) electrolytes on Au@Pd surface, as shown in Fig. S3. The frequency variation of OH stretching band in electrolytes with various cations are obviously different at the studied potentials. The frequency of OH stretching band increases in the order of Li^+^ (0.1 M) < Na^+^ (0.1 M) < K^+^ (0.1 M) < Ca^2+^ (0.1 M) < Sr^2+^ (0.1 M) < Na^+^ (5.0 M) (Fig. 3b). The Raman frequency of OH stretching vibration band is closely related to the hydrogen bonding (HB) interaction of water molecules. A higher Raman frequency of OH stretching vibration band represents weaker HB interaction, and vice versa [42, 43]. Therefore, the interfacial water in the cation electrolyte with larger ionic radius, higher valence and higher concentration shows weaker HB interaction at Pd-water interface.

Meanwhile, the coupling of OH stretching vibration with different HB interactions result in a wide OH stretching vibration band in Raman spectrum. According to the difference in HB interactions, the OH stretching vibration band can be distinguished by Gaussian fitting. As shown in Fig. 3c, d, the OH stretching vibration band is boiled down to three elements, assigning three kinds of OH stretching vibrations [1]. The lower frequency element (blue region) and middle element (orange region) are related to 4 and 2-coordinated hydrogen bonded water (4 and 2-HB^.^H_2_O), respectively. The higher frequency element (red region) is attributed to cation bonded water (M^.^H_2_O) with weak hydrogen bonding interactions. The population of M^.^H_2_O in 0.1 M Li^+^, Na^+^, K^+^, Ca^2+^, Sr^2+^, and 5.0 M Na^+^ electrolytes is 18.6%, 22.9%, 23.6%, 43.7%, 47.9%, 52.1% at –0.226 V.

To further obtain the information regarding the role that cations and water play in HER at alkaline conditions, we carried out DFT calculations to study the adsorption performance of cations (Li^+^, Na^+^, K^+^, Ca^2+^, and Sr^2+^) and water molecules on Pd (111) electrode surface. The optimized geometrical configurations of cations and water molecules are shown in Fig. 3e. In all cationic systems, HB water molecules and the hydrated cations are located at the Pd (111) surface, matching the Raman results. The Li^+^ system contains, a total of three dangling hydrogen atoms in the water molecules are closest to the Pd surface with one hydrogen atom in each interfacial water molecule pointing toward the Pd surface and another hydrogen atom forming a HB with other water molecules. The Na^+^, K^+^, and Ca^2+^ systems all contain four dangling hydrogen atoms where one of the water molecules has two dangling hydrogen atoms. In the Sr^2+^ system, there are five dangling hydrogen atoms with one water molecule sharing two dangling hydrogen atoms. We also counted the amount of water molecules between the cation and Pd (111) electrode. The number of hydrogen atoms (Fig. 3e) are four (Li^+^), six (Na^+^), seven (K^+^), seven (Ca^2+^), and eight (Sr^2+^). We also obtain the coordination number between the water molecules and cations (Fig. S4). As the cationic radius and valence increase, the coordination number of water molecules around Li^+^, Na^+^, K^+^, Ca^2+^, and Sr^2+^ are four, five, six, six, and eight according to the calculation results, which verified the increased the population of M^.^H_2_O in the in situ Raman results.

Additionally, we found that there are structural differences in the water molecule with two dangling hydrogen atoms (denoted by the purple water molecules in Fig. 3e) in the cationic systems. As the cationic radius and valence increases, the hydrogen atom that was originally far from the electrode is oriented closer to the surface forming a structure where two of the hydrogen atoms are directed toward the electrode surface (two-H down structure). We know that when the angle between the OH bond direction of water molecules and the surface normal is 52.25° (Fig. S5a), the two hydrogen atoms in the water molecule are the closest to the surface of the electrode. Therefore, we further calculate the distribution of angle of water molecules which located at the interface between the Pd surface and cation at negative potential. The angle between the OH_1_/OH_2_ bond direction and surface normal is named angle *φ*/*υ* (Fig. S5b). The average value of *φ* and *υ* angles (Table S1) gets closer to 52.25° with the ionic radius and valence increasing. These results further confirm that the structure of interfacial water can be effectively regulated by changing the cation. The AIMD simulation results also verify the structural changes of water in Li^+^, Na^+^, and K^+^ systems. A similar geometrical structure, shown in Figs. S6-S8, can be obtained for an equilibrium state region at 298 K.

**Fig. 4:**
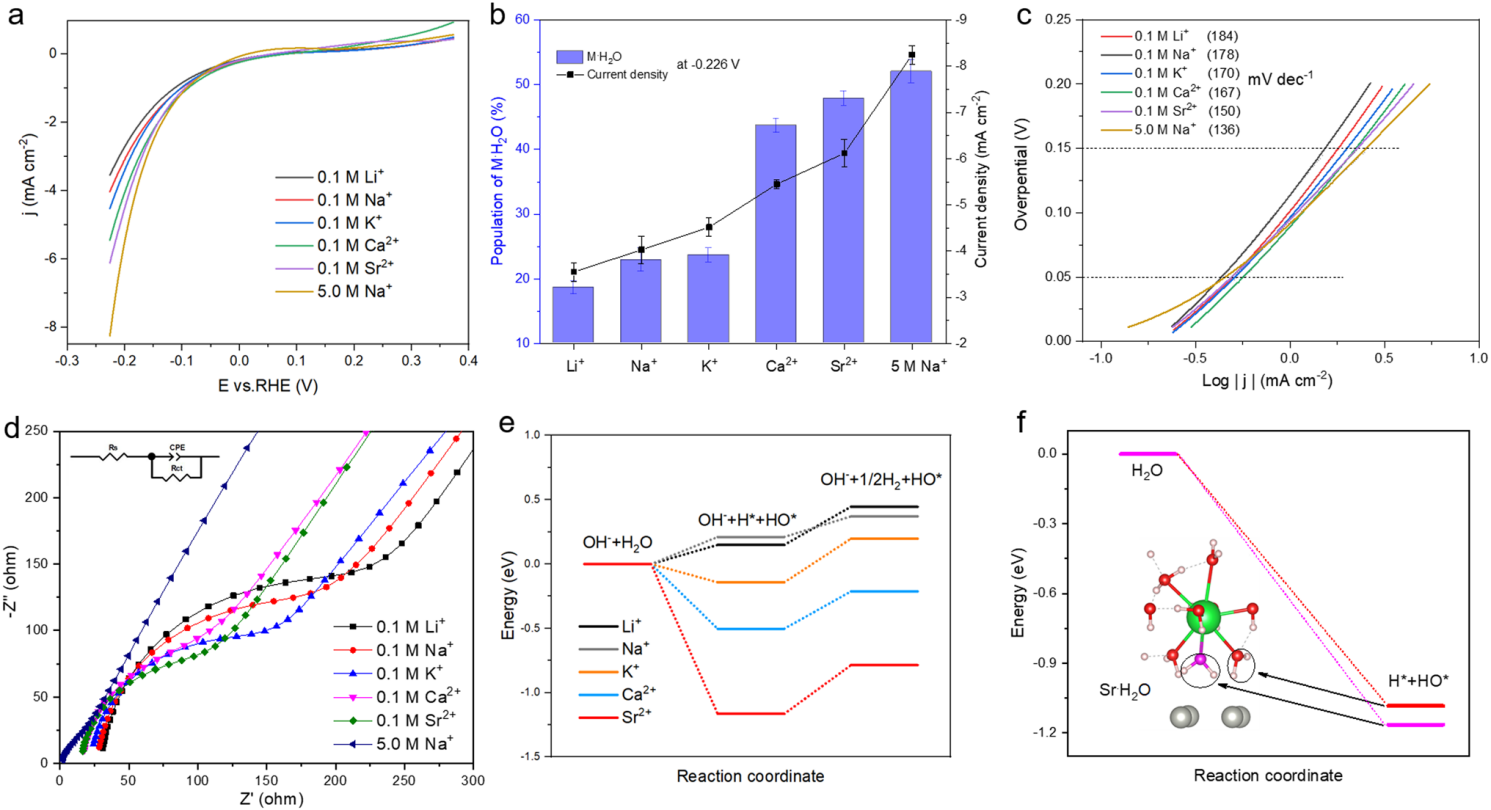
**a** HER polarization profiles on Pd polycrystalline electrode in 0.1 M MClO_4_ (pH 12, M = Li^+^, Na^+^, K^+^, Ca^2+^, and Sr^2+^) and 5.0 M NaClO_4_ (pH 12) solutions. **b** Correlation between the average values of the M^.^H_2_O populations and HER current densities at − 0.226 V in different cationic solutions at pH12 [error bars represent the standard deviation for each data point (n = 3)]. **c** Tafel slopes and **d** Nyquist plots (inset: equivalent electric circuit) of HER for Pd electrode in 0.1 M MClO_4_ (pH 12, M = Li^+^, Na^+^, K^+^, Ca^2+^, and Sr^2+^) and 5.0 M NaClO_4_ (pH 12) electrolytes. **e** Gibbs free energy profiles for the dissociation processes of water molecule without HBs on the Pd(111) surface in the Li^+^, Na^+^, K^+^, Ca^2+^, and Sr^2+^ systems. **f** Gibbs free energy profiles for the dissociation processes of water molecules with two-H down and one-H down structures on Pd(111) surface in Sr^2+^ system

Figure 4a shows the HER performance in 0.1 M MClO_4_ (pH 12, M = Li^+^, Na^+^, K^+^, Ca^2+^, and Sr^2+^) and 5.0 M NaClO_4_ (pH 12) solutions on the Pd polycrystalline electrode surface. The current density for HER is − 3.55 (0.1 M Li^+^), − 4.03 (0.1 M Na^+^), − 4.52 (0.1 M K^+^), − 5.45 (0.1 M Ca^2+^), − 6.12 (0.1 M Sr^2+^), and − 8.25 (5.0 M Na^+^) mA cm^−2^ at − 0.226 V. The HER current density measurements at − 0.226 V show a remarkably similar trend (0.1 M Li^+^  < 0.1 M Na^+^  < 0.1 M K^+^  < 0.1 M Ca^2+^  < 0.1 M Sr^2+^  < 5.0 M Na^+^) to the interfacial M^.^H_2_O populations obtained by the Raman spectroscopy measurements at –0.226 V, as shown in Fig. 4b.

We further study the Tafel slopes in different cationic solutions. The 0.05–0.15 V overpotential range was selected to calculate the Tafel slopes, as shown in Fig. 4c. The HER Tafel slopes are 184, 178, 170, 167, 150, and 136 mV dec^−1^ with a trend of 0.1 M Li^+^  < 0.1 M Na^+^  < 0.1 M K^+^  < 0.1 M Ca^2+^  < 0.1 M Sr^2+^  < 5.0 M Na^+^, indicating that the HER kinetic process can be accelerated by changing the cationic radius, valence, and concentration. Meanwhile, the electrochemical impedance spectroscopy (EIS) is used to study solution resistance. The EIS was performed in constant potential mode at −0.1 V vs RHE (0.1 V overpotential) over a frequency range from 100 kHz to 0.1 Hz, matching the potential range of Tafel slopes. Figure 4d shows the fitting curve for Nyquist plot and corresponding equivalent electric circuit. The semicircle diameter results indicate that the solution resistance follows this order: 0.1 M Li^+^  > 0.1 M Na^+^  > 0.1 M K^+^  > 0.1 M Ca^2+^  > 0.1 M Sr^2+^  > 5.0 M Na^+^.

The same trend of HER current density at − 0.226 V, Tafel slopes, solution resistance, and interfacial M^.^H_2_O populations well confirm that the HER performance was vastly improved as the cation radius, valence, and concentration increase. The DFT results also point that the cations with higher valence states and larger ionic radii will produce more water molecules with the two-H down arrangement at the Pd surface. Therefore, the water molecules with two-H down structure can accelerate the HER rate.

In order to further understand the regulatory role of cationic water molecules in the HER process, we also calculated the Gibbs free energy for dissociation reaction of interfacial water on the Pd (111) surface for the different cation systems (Fig. 4e). Water molecules that are close to the surface and have at least one hydrogen atom toward the surface do not form hydrogen bonds with other water molecules. And the computational hydrogen electrode model has further calculated the HER reaction process for the surface hydrogen that originates from the dissociation of H_2_O. The Gibbs reaction energies for Li^+^, Na^+^, K^+^, Ca^2+^, and Sr^2+^ are calculated to be 0.30, 0.16, 0.34, 0.29, and 0.38 eV, respectively. Although the Gibbs reaction energy for generation of H* in Sr^2+^ system is only slightly higher than that of others cations, a high HER performance can still be observed due to the high concentration of surface H* and a low adsorption energy of H* (− 0.33 eV, the adsorption energy is calculated by *E*_ads_ = *E*_tot_−*E*_H_−*E*_metal_ (*E*_ads_ is adsorption energy, E_tot_ is total energy of adsorbed structures, *E*_H_ is the energy of H*, *E*_metal_ is the energy of structure without H*) in Sr^2+^ system. Therefore, the activity of HER shows a better performance in higher valence and larger radius cationic electrolyte.

The dissociation process of water molecules with different structures was also investigated. We calculated the Gibbs free energy of water for the H_2_O to OH* and H* process in the Sr^2+^ electrolyte system, as shown in Fig. 4f. The water molecule with more downward structure (purple H_2_O molecule) shows a lower free energy than its neighboring water molecule. Therefore, combining the electrochemistry, in situ Raman, and the Gibbs energy calculation results, the increased population of interfacial water with two-H down structure can accelerate the processes involved in the HER.

## Conclusions

The electrochemical SERS spectra of interfacial water on Pd surfaces in electrolytes containing different cations were collected. SERS spectra of the OH stretching vibration band reflect the behavior of interfacial water via potential/cation-dependent frequency, intensity, and local structures. Combining experimental and simulated results, we found that the cations could optimize the structure of the interfacial water by increasing the number of H down structures, thus improving the efficiency of charge transfer between water and electrode to enhance the HER activity under the bias potential. We discovered that the HER activity is proportional to the radius, valence, and concentration of the cation in the electrolyte. In this respect, interfacial cations change the transport paths of reactants and products during HER process improving the overall reaction rate. The discoveries presented in this study, coupled with the established experimental and theoretical capacities, unveil a thrilling prospect for future research in various energy conversion domains. These strategies of cation tuning hold the potential to decipher the structure of interfacial water in electrocatalytic reactions and enhance the overall reaction rates of such systems, extending their applicability to diverse energy conversion fields.

## Supplementary Information

Below is the link to the electronic supplementary material.

Supplementary file1 (PDF 875 kb)
