# Supplementary material for: Exploring the Cation Regulation Mechanism for Interfacial Water Involved in the Hydrogen Evolution Reaction by In Situ Raman Spectroscopy
Source: Nanomicro Lett. 2023 Dec 18;16:53. doi: 10.1007/s40820-023-01285-1 (PMC10728385; doi:10.1007/s40820-023-01285-1)
Supplement: Supplementary file 1 — Supplementary file1 (PDF 875 kb) [file 40820_2023_1285_MOESM1_ESM.pdf]

Supporting Information for

Exploring the Cation Regulation Mechanism for Interfacial Water Involved in the Hydrogen Evolution Reaction by in situ Raman Spectroscopy

Xueqiu You[1], Dongao Zhang[2], Xia-Guang Zhang[3], Xiangyu Li[1], Jing-Hua Tian[4], Yao-Hui Wang[2,*], Jian-Feng Li[2,4,*]

[1]School of Ocean Information Engineering, Fujian Provincial Key Laboratory of Oceanic Information Perception and Intelligent Processing, Jimei University, Xiamen 361021, P. R. China

[2]State Key Laboratory of Physical Chemistry of Solid Surfaces, MOE Key Laboratory of Spectrochemical Analysis and Instrumentation, iChEM, College of Chemistry and Chemical Engineering, College of Materials, College of Energy, Xiamen University, Xiamen 361005, P. R. China

[3]Key Laboratory of Green Chemical Media and Reactions, Ministry of Education, Collaborative Innovation Center of Henan Province for Green Manufacturing of Fine Chemicals, College of Chemistry and Chemical Engineering, Henan Normal University, Xinxiang 453007, P. R. China

[4]Innovation Laboratory for Sciences and Technologies of Energy Materials of Fujian Province (IKKEM), Xiamen 361005, P. R. China

[*]Corresponding author. E-mail: Li@xmu.edu.cn (Jian-Feng Li); yaohuiwang@xmu.edu.cn (Yao-Hui Wang)

Supplementary Figures

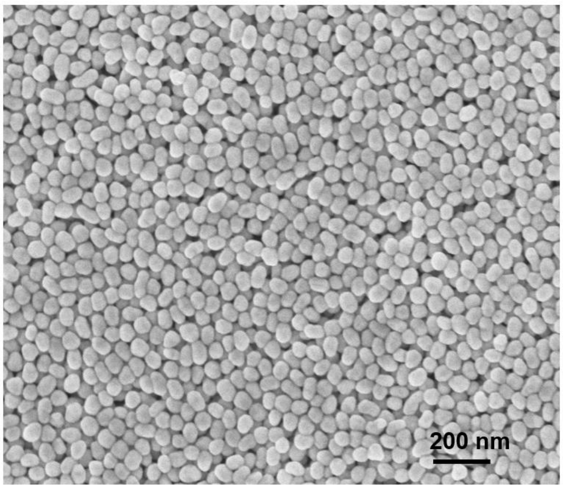

Fig. S1 SEM image of Au NPs

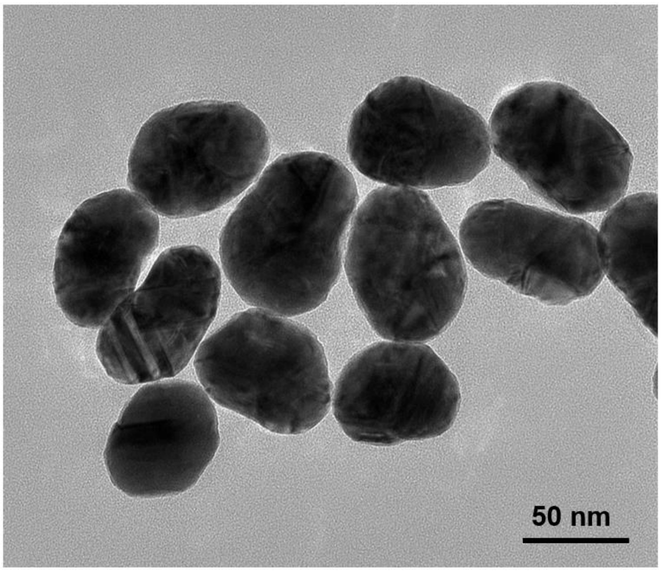

Fig. S2 TEM of image of Au@Pd NPs

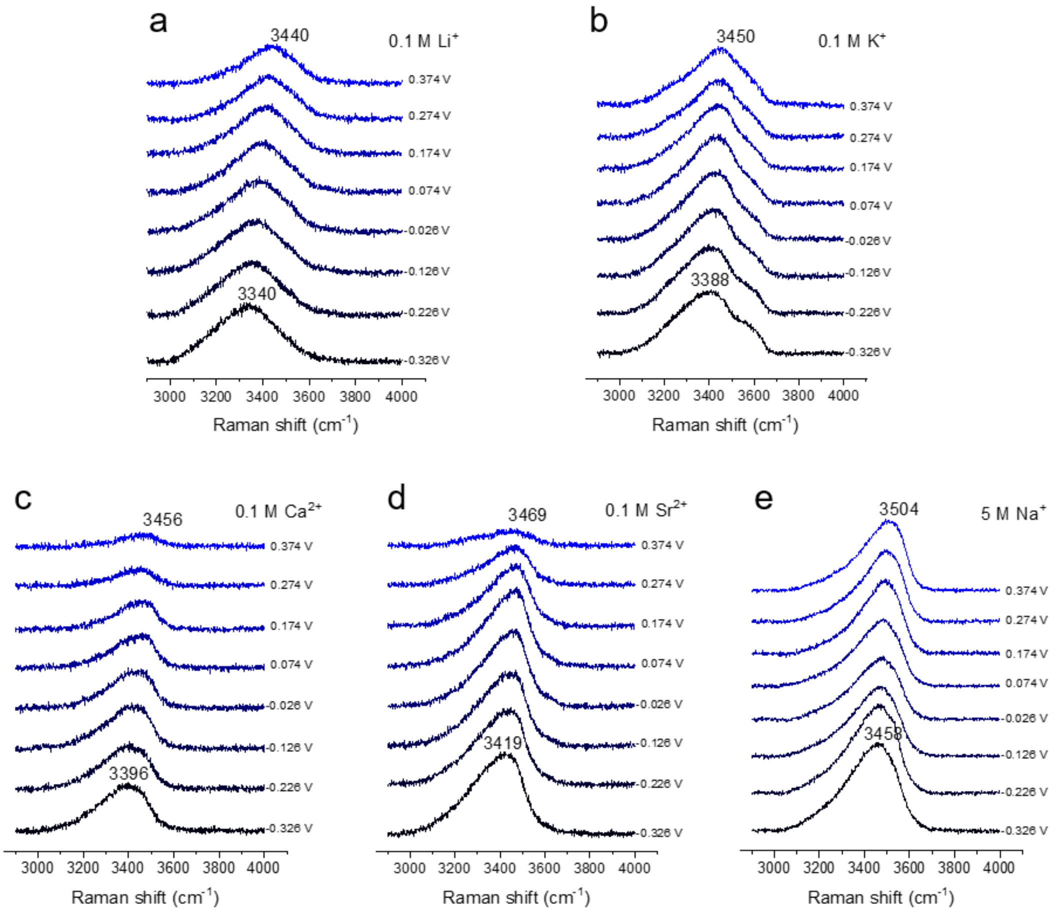

Fig. S3 In situ Raman spectra of interfacial water on Au@Pd surface in alkaline electrolytes solutions (pH 12) containing **a** 0.1 M $LiClO_4$, **b** 0.1 M $KClO_4$, **c** 0.1 M $Ca(ClO_4)_2$, **d** 0.1 M $Sr(ClO_4)_2$, and **e** 5 M $NaClO_4$

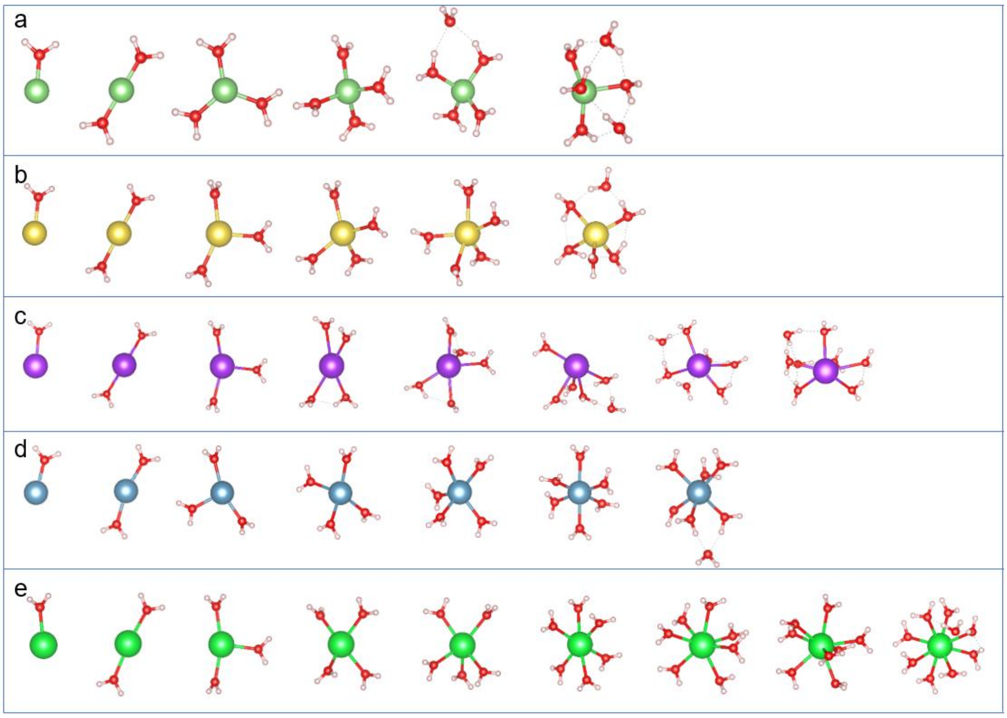

Fig. S4 Geometrical structure of cations (**a-e**: Li^+, Na^+, K^+, Ca^{2+}, and Sr^{2+}) and water molecule with various coordination number

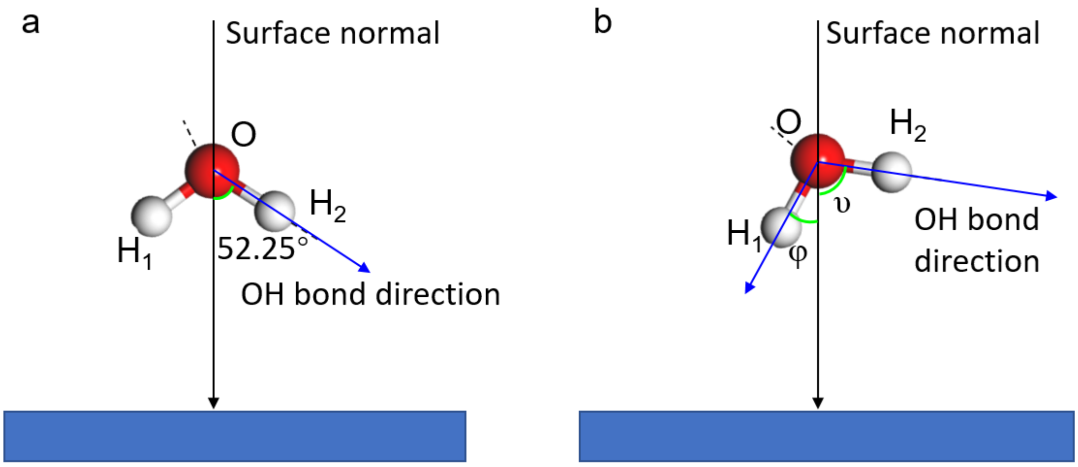

Fig. S5 The angle (**a**: 52.25°; **b**: φ / υ angles) between the OH bond direction of water molecules and the surface normal

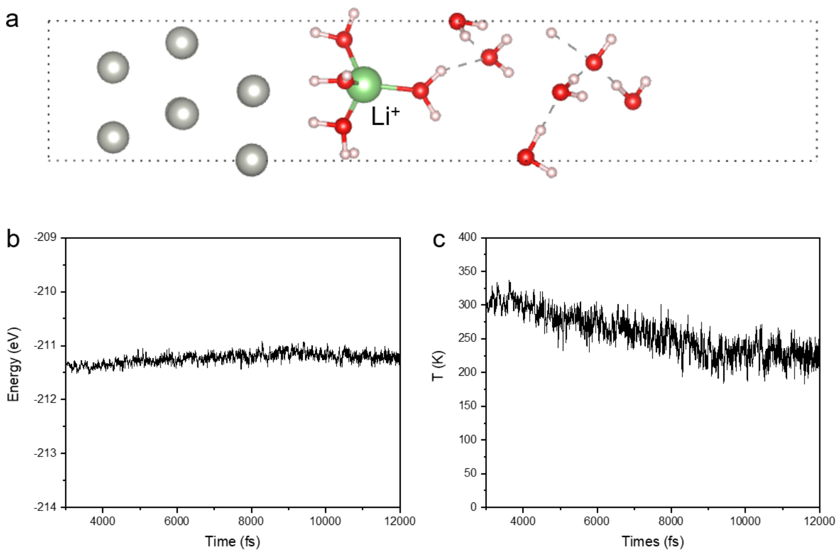

Fig. S6 AIMD results of Li^+ system showing **a** a snapshot of a typical of $Li^+ – H_2O$ structure on a Pd(111) surface along with the **b** energy and **c** temperature curves

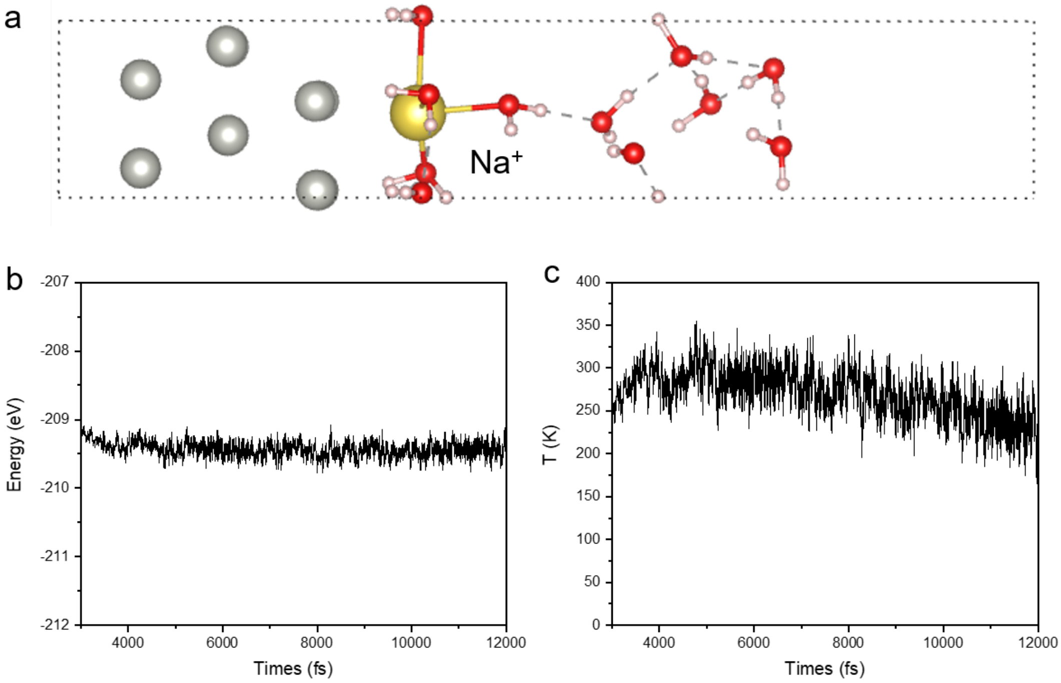

Fig. S7 AIMD results of Na^+ system showing **a** a snapshot of a typical $Na^+ – H_2O$ structure on the Pd(111) surface along with the **b** energy and **c** temperature curves

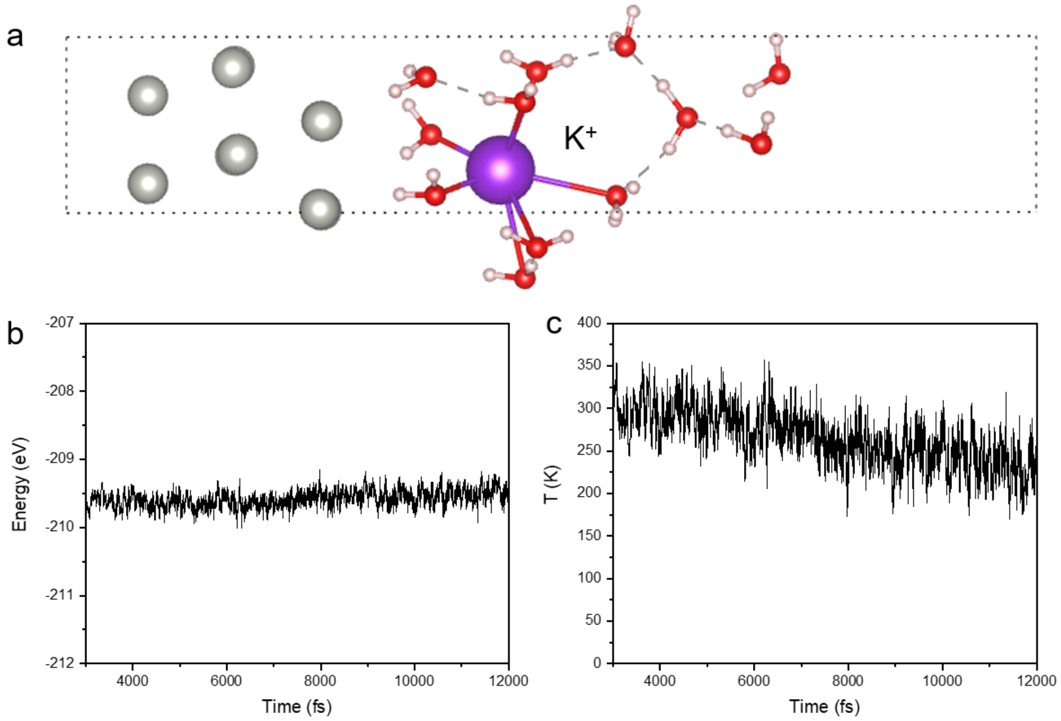

Fig. S8 AIMD results of K^+ system showing **a** a snapshot of a typical structure of K^+ – H_2O structure on the Pd(111) surface along with the **b** energy and **c** temperature curves

Table S1 The distribution of φ and υ angles

	Angle distribution										Ave.	
	H_2O-1		H_2O-2		H_2O-3		H_2O-4		H_2O-5			
	φ	υ	φ	υ	φ	υ	φ	υ	φ	υ	φ	υ
Li^+	4.35	127.07	20.18	101.48	20.30	87.98					14.94	105.51
Na^+	13.23	115.07	16.09	106.54	24.76	89.20	25.43	76.51	29.39	69.97	21.78	91.46
K^+	15.75	135.22	23.50	95.79	24.83	84.12	27.78	78.22	31.14	72.04	24.60	93.08
Ca^{2+}	12.86	126.75	16.43	120.27	25.35	90.30	32.38	86.34	42.36	53.60	25.88	95.45
Sr^{2+}	15.31	125.57	15.32	94.81	20.45	86.54	23.76	78.66	40.98	55.04	23.16	88.12

Supplementary Notes

Electromagnetic field simulation

The finite element method (FEM) was widely used to investigate the near and far-field

optical properties of the plasmonic characteristics, such as electromagnetic field enhancement, light scattering, and light absorption. FEM simulations were performed using a commercial software (COMSOL Multiphysics) to probe the electromagnetic field distribution of Au@Pd, which was modeled with a heptamer on the Pd substrate. The perfectly matched layer was applied to all the simulation boundaries for absorbing incident light with minimal reflections. Specifically, the Au core is 55 nm in diameter with a 1-nm thick Pd shell, and the interparticle distance is set to 0 nm. The refractive index of background was set to 1.33, corresponding to the surrounding water in the system. The frequency dependent optical constant of Au and Pd was obtained from literature references [S1, S2].

Supplementary References

[S1] P. Johnson, R. Christy. Optical constants of transition metals: Ti, V, Cr, Mn, Fe, Co, Ni, and Pd. Phys. Rev. B **9**(12), 5056-5070 (1974). https://doi.org/10.1103/PhysRevB.9.5056

[S2] P. B. Johnson, R. W. Christy. Optical constants of the noble metals. Phys. Rev. B **6**(12), 4370-4379 (1972). https://doi.org/10.1103/PhysRevB.6.4370
